# Stability of Nanocrystalline Spark Plasma Sintered 3Y-TZP

**DOI:** 10.3390/ma3020800

**Published:** 2010-01-28

**Authors:** Ravikiran Chintapalli, Alvaro Mestra, Fernando García Marro, Haixue Yan, Michael Reece, Marc Anglada

**Affiliations:** 1 Center for Structural Integrity and Reliability of Materials, Department of Materials Science and Metallurgical Engineering, Universitat Politècnica de Catalunya, Av. Diagonal 647, 08028 Barcelona, Spain; E-Mails: alvaro.mestra@upc.edu (A.M.); fernando.garcia.marro@upc.edu (F.G.M.); 2 School of Engineering and Materials Science, Queen Mary College, University of London, Mile End Road, London E1 4NS, UK; E-Mails: h.x.yan@nanoforce.co.uk (H.Y.); m.j.reece@nanoforce.co.uk (M.R.); 3 Nanoforce Technology Ltd, Mile End Rd, London E1 4NS, UK

**Keywords:** 3Y-TZP, grain size, degradation, phase transformation

## Abstract

Spark plasma sintered 3Y-TZP has been investigated with respect to hydrothermal ageing and grinding. The sintering was performed between the temperatures of 1,100 and 1,600 °C for a soaking time of 5 minutes and the resulting materials were obtained with grain sizes between 65 to 800 nm and relative densities between 88.5 to 98.8%. Experiments on hydrothermal ageing in water vapour at 131 °C, 2 bars during 60 hours shows that phase stability is retained, elastic modulus and hardness of near surface region measured by nanoindentation does not change in fine grain (<200 nm) materials, in spite of porosity. In ground specimens, very small amount of transformation was found for all grain sizes studied.

## 1. Introduction

Yttria doped zirconia ceramics are very attractive candidates for biomedical applications and are commonly used in hip implants and dental restorations. They have excellent mechanical properties, good wear resistance and bio-compatibility. Their fracture toughness is enhanced by the activation of the tetragonal to monoclinic (t-m) phase transformation in the region in front of the crack tip (transformation toughening), which is accompanied by an increase in volume [1].

A critical and widely discussed characteristic of 3% mol yttria doped tetragonal zirconia polycrystals (3Y-TZP) is the phenomenon of low temperature degradation (LTD) or hydrothermal degradation in air and aqueous environments at relatively low temperatures. This effect was first observed by Kobayashi *et al.* [2] at temperatures close to 250 °C in air and it is widely documented in the literature [3,4,5,6,7,8]. Degradation activates surface t-m transformation and deteriorates surface mechanical properties. Close to room temperature, transformation proceeds at very slow rates, but during many years it has been thought that this effect was negligible at the human body temperature. However, recently there have been many reports which document the existence of monoclinic phase at the surface of explanted femoral heads several years after implantation [9,10,11]. The study of LTD in medical grade zirconia at human corporal temperature is usually carried out *in vitro* at higher temperatures in an autoclave [12].

In principle one method to reduce LTD in 3Y-TZP is to refine the grain size in order to decrease the difference in chemical free energy between t and m-phases [4]. The effectiveness of this approach has been clearly demonstrated for grain sizes of the order to the micron, although LTD is still present in dense 3Y-TZP with the smallest grain size achieved by conventional sintering. By contrast, reducing the grain size in the nanometric scale in sintered bodies has been hardly studied because of the difficulty of achieving this grain size after sintering. Some researchers reported nanometric grain sizes can be obtained by conventional sintering [13,14,15,16] although the final grain size depends on the starting crystallite size and sintering cycle. Nanometric grain sizes were achieved by long sintering times at low temperatures [16]. But studies of hydrothermal degradation on nanometric grain sizes were very limited.

However, the effect of decreasing the particle size in free powder on LTD has been studied by Djurado *et al.* [17] and by Schubert and Frey [6], who found that degradation was stopped if the particle size was reduced to the nanometric scale. In conventionally sintered bodies with average grain size usually equal or larger than 0.3 µm, degradation is faster than in free particles [18,19,20]. Here, sintered bodies of 3Y-TZP have been prepared by spark plasma sintering (SPS) with a range of grain sizes between 65 and 800 nm by sintering at different temperatures between 1,100 and 1,600 °C. Resistance to LTD and mechanical properties before and after degradation have been determined and analyzed in this grain size range.

## 2. Experimental

### 2.1. Materials

Commercially available yttria stabilised zirconia powder (Tosoh Co, Japan) with 3% molar of yttria was used to prepare the samples. The mean crystallite size of the powder is ≈29 nm. The powder was sintered using spark plasma sintering (SPS FCT HP D25I, FCT system GmBh) at temperatures between 1,100 °C and 1,600 °C for 5 minutes at a pressure of 100 MPa. For comparison, 3Y-TZP specimens were also conventionally compacted and sintered at 1,450 °C for 2 hours in the absence of pressure and these samples will be referred as “AS-300” further in the paper.

### 2.2. Experimental

The samples prepared by SPS were in the form of discs of 20 mm diameter and 4 mm thickness. The samples sintered by conventional method were cylindrical bars (ø 10 mm and 70 mm long) which were cut with a diamond saw into discs of 2 mm thickness. They were polished in different steps using 30, 6 and 3 µm diamond paste and finally with 0.03 µm colloidal silica. The surface roughness of the samples was measured in the range of *R_a_*≈0.02–0.05 µm using laser scanning confocal microscopy (Olympus, LEXT OLS 3100). Density of all samples was determined using Archimedes principle. Table 1 shows the detailed description of samples with listed properties. In order to observe the microstructure, one polished sample for each condition was thermally etched in air in order to reveal the grain boundaries. Thus, one sample each of the SPS-65, SPS-90 and SPS-120 were subjected to a 1-hour thermal treatment at 1,000 °C, while one SPS-800 sample was thermally etched at 1,500 °C and a AS-300 sample was treated at 1,350 °C. The microstructure of the samples was observed using a Jeol JSM-7001F field emission scanning electron microscope (SEM) and SPS 800 was observed by using a FIB-SEM dual work station (ZEISS NEON® 40). Mean grain size was measured using linear intercept method by counting at least 250 grains.

A group of samples were subjected to hydrothermal ageing in an autoclave at 131 °C for 60 hours in contact with water vapour under a pressure of 2 bars. Aged samples will be referred as 60H. Another group of samples were subjected to wet grinding in the presence of water using rough diamond grit (120 µm) under pressure of 0.2 MPa for 3 min at 300 rpm of the spindle.

All the samples were analysed by X-ray diffraction with a Bruker AXS D8 diffractometer by using Ni-filtered Cu-K_α_ radiation. Samples subjected to hydrothermal degradation and grinding were analysed by using Bragg-Brentano θ/2θ configuration with incidence angle 12° and grazing incidence angle 2° respectively. The samples will be referred further in the article with the reference code, as shown in the Table 1. Phase transformation was quantified in terms of monoclinic volume fraction (*V_m_*) calculated with the following expression proposed by Toraya *et al.* [21]:

(1) $$V_{m}\;=\;\frac{1.311X_{m}}{1+0.311X_{m}}$$

Hardness (HV1) values were measured with 1 kg Vickers indentations. For estimating the indentation facture toughness, 20 kg Vickers indentations were performed and the equation used for its determination was that proposed by Anstis *et al.* [22] for median cracks, which is given by:

(2) $$K_{Ic}=\xi \sqrt{\frac{E}{H}}\frac{P}{c^{3/2}}$$

where *P* is the applied load, *E* is the Young’s modulus, *H* is the hardness and *c* is the length of the surface trace of the half penny crack measured from the centre of the indent. ξ is an empirically determined calibration constant, taken to be 0.016 ± 0.004 based on a fit to experimental data using independent fracture toughness measurements.

In addition, contact hardness and elastic modulus were determined by nanoindentation with a Berkovich tip up to 1.5 µm depth. The Berkovich tip was calibrated against a standard fused silica. Nanoindentation tests were performed with an MTS Nanoindenter XP with a continuous stiffness measurement (CSM) module, which allows continuous measurement of the contact stiffness *S* and load *P* as a function of penetration depth *h*. Indentations were made to a maximum load of 500 mN to a maximum penetration depth of about 1600 nm, and under a constant drift rate of 0.05 nm/s. Nine indentations were made as 3x3 matrices on each material and the results were presented with standard deviation. The values of hardness and Young’s modulus were calculated as a function of penetration depth using the method of Oliver and Pharr [23]. In this method, the contact depth is estimated from the load-displacement data using:

(3) $$h_{c}=\;h{\;}_{\max}-\varepsilon \frac{P_{\max}}{S}$$

where *P*_max_ is the peak indentation load and ε is a constant which depends on the indenter geometry [23]. Empirical studies have shown that ε≈0.75 for a Berkovich indenter. From the measurements of load and displacement data, the projected contact area, *A,* of the indentation impression is estimated by evaluating indenter shape function at the contact depth, *h*_c_; that is *A=f*(*h*_c_). The shape function, *f(d)*, relates the cross-sectional area of the indenter to the distance, *d*, from its tip. Once the contact area is determined from the load displacement data, the hardness and effective elastic modulus, *E*_eff_, were calculated as follows:

(4) $$H=\;\frac{P_{\max}}{A}$$

(5) $$E_{eff}\;=\;\frac{\sqrt{\pi }}{2\beta }\;\frac{S}{\sqrt{A}}$$

where β depends on the indenter tip geometry and is equal to 1.034 for a Berkovich tip. This technique provides data with continuous measurement of the hardness and elastic modulus as a function of depth. With due care and analysis, the hardness and elastic modulus of many materials can be measured using these methods with accuracies of better than 10% [24].

## 3. Results

### 3.1. Microstructure and Material Properties

Table 1 shows the sintering temperature, density and grain size of all samples. Because of the small starting crystallite size and good sintering activity, moderate to fully dense materials were obtained using the SPS technique. A fully dense material was also obtained with conventional sintering. Higher densities were achieved with higher sintering temperature. Grain growth is also evident as the sintering temperature increases. Very fine grain materials were obtained at low temperatures compared to conventional sintering.

**Figure 1:**
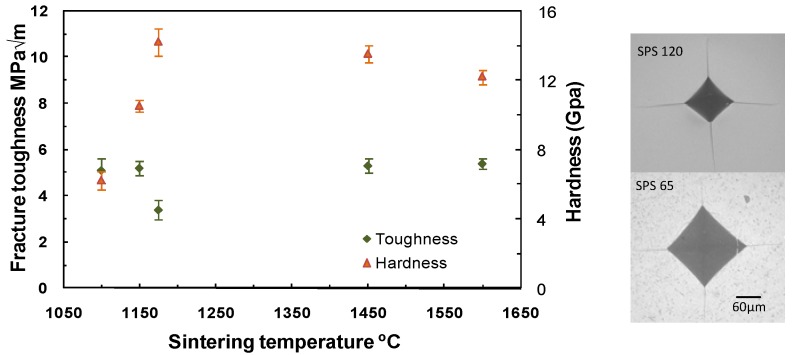
Toughness (left axis) and hardness (right axis) against sintering temperature, Vickers indentations (20 kg).

Figure 1 displays the indentation fracture toughness and hardness against sintering temperature; also pictures of cracks emanating from Vickers imprints are shown. Indentation toughness and hardness are average values calculated from four indentations on each sample. The material with high density and fine grain size (SPS-120) has higher hardness but relative lower toughness as compared to specimens with larger grain size and similar density (SPS-800 or AS-300). On the other hand, relatively low dense specimens (SPS-65 and SPS-90) have low hardness, but, surprisingly, higher indentation fracture toughness than dense SPS-120.

**Table 1:** Conditions and measured properties of the different specimens.

	Reference	SPS-65	SPS-90	SPS-120	SPS-800	AS-300
Properties						
Sintering Temp. (°C)		1100	1150	1175	1600	1450
Density(g/cm^3^)		5.42	5.71	6.00	6.05	6.10
Mean Grain Size (nm)		65	90	120	800	300
Elastic modulus (GPa)		121 ± 5	200 ± 7	230 ± 3	223 ± 2	231 ± 3

**Figure 2:**
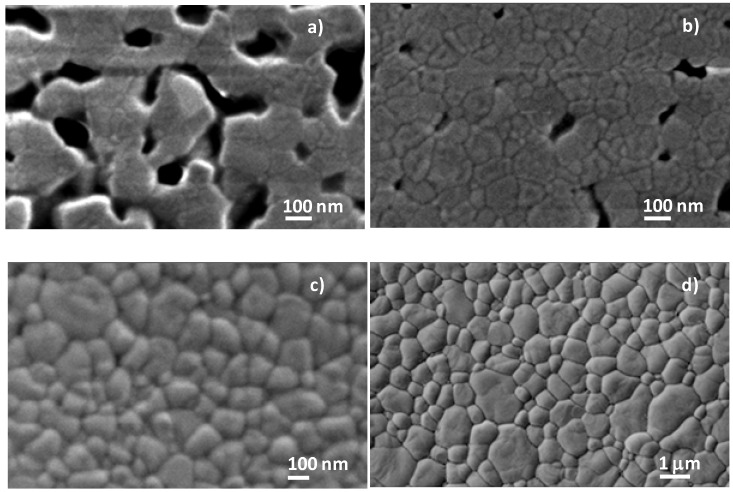
Microstructures of thermally etched SPS sintered samples observed in SEM: (a) SPS-65; (b) SPS-90; (c) SPS-120; and (d) SPS-800.

The mean grain size of SPS-800 (specimen sintered at high temperature) is 800 nm and by looking at the microstructure (see Figure 2), it can be observed that there is no porosity and that the grains appear with sharp edges. In SPS-120, grains appear to be more with a rounded shape, suggesting also a dense material as confirmed by density measurements. In SPS-90 and SPS-65, the grains have a round shape and many pores can be seen on the surface. This is because of the poor sintering conditions, since the soak times at 1,100 and 1,150 °C were too short. When the soaking temperature was between 1,100 and 1,200 °C for 5 minutes the grain size was smaller than obtained in conventional sintering (around 300 nm). This suggests that high soaking temperatures will accommodate grain growth, at least in SPS process [25].

### 3.2. Hydrothermal Degradation

**Figure 3:**
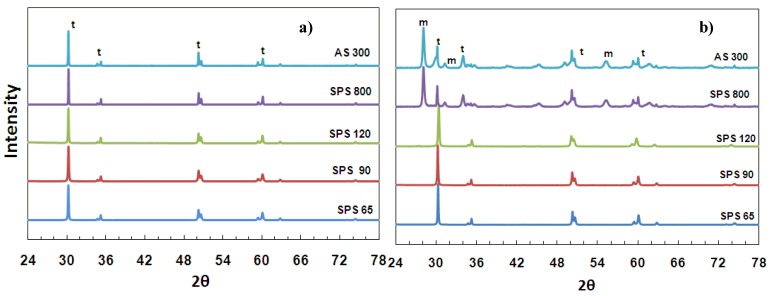
X-ray diffraction patterns of samples: a) after sintering and fine polishing; b) after 60 hours autoclave ageing. (t-tetragonal, m-monoclinic).

Figure 3 (a) shows the X-ray diffraction patterns of all samples after sintering and fine polishing. The material is purely tetragonal and no monoclinic phase is found. Figure 3 (b) displays the XRD patterns of samples after 60 hours of ageing in autoclave at 131 °C (60H samples). It is observed that the stability of the tetragonal phase is retained after hydrothermal ageing in samples with fine grain size (SPS-65, SPS-90 and SPS-120), as no monoclinic peaks were found. The remarkable fact is that, in spite of the high porosity of the specimen with the smallest grain size (SPS-65), which was sintered at the lowest temperature is fully resistant to LTD.

By contrast, the tetragonal phase is destabilized and transformed to monoclinic in samples with larger grain size (SPS-800 and AS-300). From Figure 3 (b), monoclinic peaks can be observed at 2θ values of 28.1°, 31.4° and 55.8°. The amount of phase transformation (t-m) was calculated using equation (1). Large amount of monoclinic volume fraction, 65.4 and 71.4%, were detected in specimens of grain sizes of 300 and 800 nm, respectively.

**Figure 4:**
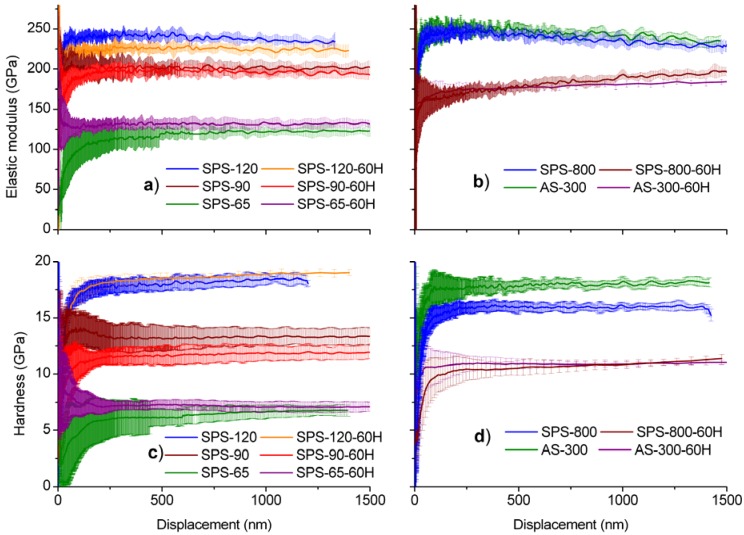
Elastic modulus and hardness as a function of penetration depth: (a) & (c) for fine grain materials, (b) & (d) for coarse grain materials respectively.

Elastic modulus and hardness were determined by nanoindentation using a Berkovich indenter and a continuous stiffness measurements unit and the results are shown in Figure 4. Indentations were made before and after hydrothermal ageing. Both magnitudes were found to change widely in the specimens before ageing because of their different porosity. However, there is a clear correlation between density and contact hardness and elastic modulus in specimens SPS-65, SPS-90 and SPS-120: elastic modulus and contact hardness diminish by increasing porosity.

After hydrothermal degradation, elastic modulus and contact hardness decrease only in specimens with larger grain size, but they remain unchanged in specimens with grain size equal or smaller than 120 nm. Figure 4 shows elastic modulus and contact hardness for fine grain materials (≤120 nm) and coarse grain materials (≥300 nm). It can be appreciated that, in coarse grain materials, ageing induces a large decrease in elastic modulus and contact hardness. A closer inspection of the curves of aged specimens that suffer LTD reveals that, after a certain penetration depth, the elastic modulus and hardness tend to recover their values for non-aged specimens because of the effect of the healthy non degraded material beneath the affected surface layer.

### 3.3. Grinding

**Figure 5:**
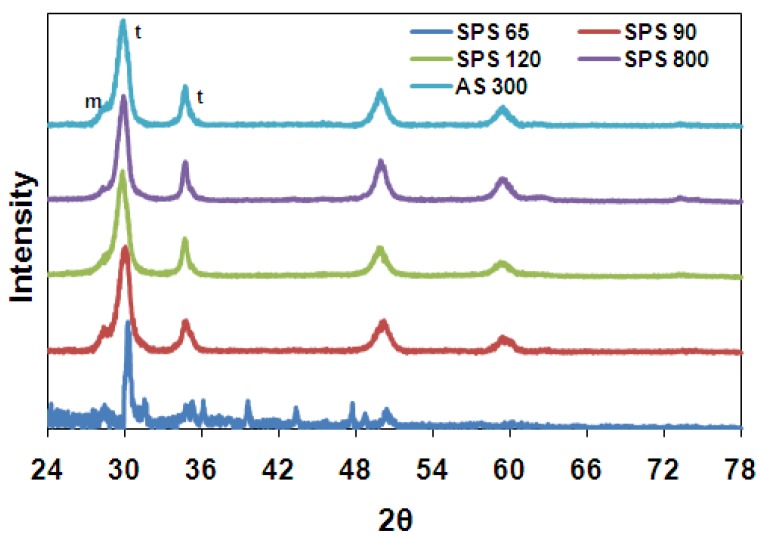
X-ray diffraction patterns of samples after grinding.

The wet ground samples were ultrasonically cleaned in acetone for 15 minutes. All the ground samples were observed under grazing incidence X-ray diffraction at an angle of 2°. This was done in order to measure a higher amount of monoclinic phase by concentrating the measurement in a shallow depth layer with respect to common X-rays diffraction configuration. From previous experience on 3Y-TZP with 0.3 μm grain size, it was known that under the grinding conditions used here the amount of monoclinic was low and decreasing rapidly with depth. Figure 5 shows the XRD patterns obtained. The sample SPS-65 which has high porosity was contaminated by some foreign material into its pores during grinding, probably from the grinding disc. The unusual peaks that are observed for this sample in Figure 5 belong to the foreign material, but these peaks did not overcome the zirconia peaks. Only a small raising monoclinic peak can be seen in all ground samples, in spite of using grazing incidence.

**Figure 6:**
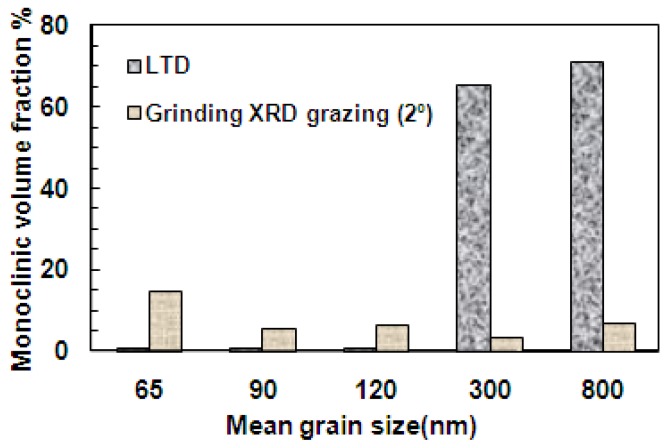
Monoclinic volume fraction in relation to the grain size.

Also a small displacement of peaks can be observed due to grinding induced residual stresses. Under high loads and grinding speeds, a small amount of t-m transformation is found in all materials irrespective of grain size (Figure 6). No significant differences are observed in the amount of transformation among the samples under present conditions with the exception of SPS-65 which is slightly more transformed than others; we believe this is due the stronger contact between grinding particles and surface since there is less contact area between the grinding grit and porous surface of the specimens. Also there may be also stronger interaction between grinding particles and pores. In summary, in the range of grain sizes studied, grain size does not strongly affect t-m transformability under stress. On the contrary, transformability under water vapour is strongly affected by grain size in the range studied here.

## 4. Discussion

It is well known that long soak time has a great influence on the density in SPS [26,27]. The soak time of 5 minutes used here is too short for obtaining a high density material at the lower temperatures used, but for temperatures equal or higher than 1,175 °C relatively high density and small grain size are achieved. Materials will be porous (relative density between 61–90% of theoretical density) when the sintering temperature is below 1,050 °C and the soak time is limited to 15 minutes, but dense materials are achieved only when the soak time is above 60 minutes [26]. Under the present conditions, porous to dense materials were obtained between temperatures 1,100 and 1,600 °C due to limited soaking time (5 minutes). The densification mechanisms were proposed by Bernard-Granger *et al.* for 3Y-TZP sintered by SPS [26].

Porosity is obviously the main reason for the low Vickers hardness and contact Berkovich hardness (in SPS-65, Vickers hardness drops to one third of its value for dense AS-300 specimens) while the elastic modulus is only reduced to about half. These changes have an influence on the measured indentation fracture toughness from the lengths of cracks emanating from Vickers indents (Lawn *et al.* [28]; Anstis *et al.* [22]).

It is well known that the determination of indentation fracture toughness is based on the elastic-plastic behaviour under the indent that was modelled assuming that a median/radial crack system is created due to tensile stresses that form during unloading. As has been commented by many authors, see, for example, recent comments of Kruzica *et al.* [29], the standard deviation on the fit to obtain this calibration constant is large even if only well behaved materials are considered. In the case of transformable 3Y-TZP with large grain size, Vickers cracks have the shape of Palqmvist cracks [30] so the application of equation (2) is questionable, however we have used because in specimens with nanometric grain size, it is not clear whether indentation cracks are also Palmqvist or median cracks.

In addition, indentation of low porosity material produces not only plastic deformation but also it may induce compaction of the volume underneath the contact area so that there is a reduction in the driving force for cracks to grow from the residual elastic strain field after unloading. This may be the main reason for the larger indentation fracture toughness encountered in the most porous specimen as compared to dense materials. That is, the porous material is not tougher; instead it is the residual indentation stress field that is weaker. The reason for finding higher values of indentation fracture toughness by using equation (2) originates by the increase in (E/H) in the most porous specimens with respect to the dense material (about 1.5). Indentation fracture toughness decreases in dense materials when the grain size is reduced as can be appreciated by comparing AS-300 and SPS-120. Recently the fracture strength of 3Y-TZP has been studied for a range of grain sizes between 110 and 480 nm by Eichler *et al.* [31]. It was shown that biaxial fracture strength decreases with the grain size and that the fracture strength is governed by the concurrent change in fracture toughness. Therefore the increase in indentation fracture toughness for the smaller grain size is believed to be caused by the use of Equation (2).

Under the conditions of hydrothermal degradation studied here, LTD does not occur for specimens with grain sizes equal or smaller than 120 nm. In materials with conventional grain size in which degradation takes place, it is well known that porosity increases degradation as has been reported by Masaki, T. [32]. Here, in nanometric grain size specimens, since there is no degradation by water species, the presence of porosity has no effect on degradation of the surface.

By preparing sections perpendicular to the surface by focused ion beam machining and observation by SEM on degraded 3Y-TZP, Gaillard *et al*. [33] have shown the existence of microcracks mostly parallel to the surface which has been associated to the drop in elastic modulus in aged specimens, because of a reduction in contact stiffness. By contrast, in our fine nanometric grain size specimens, since there is no degradation of the surface, no reduction of elastic modulus and hardness is detected by nanoindentation [see Figure 4a) and Figure 4c)].

Eichler *et al.* [31] have recently studied hydrothermal degradation of 3Y-TZP with grain sizes in the range of 110–480 nm and also fracture toughness in 2Y-TZP with average grain sizes between 150 and 900 nm [34]. Although their degradation tests used shorter times and higher temperature and vapour pressure, their results are similar to ours in the sense that significant degradation occurred only in specimens with grain sizes larger than 210 nm. Only very small amount of monoclinic phase was found by these authors in sintered bodies with 110 nm grain size. With respect to fracture toughness, they found that in 2Y-TZP (which is more transformable than 3Y-TZP) both, that the extent of stress induced tetragonal to monoclinic phase transformation at the crack flanks and the fracture toughness, diminished by decreasing the grain size with the exception of the largest grain-size sample studied (900 nm).

Though the mechanism of low temperature degradation is still at debate, it is agreed that water species from moisture environment penetrate into the tetragonal lattice and occupy oxygen vacancies during hydrothermal ageing. These oxygen vacancies are annihilated near the surface and their concentration could be low enough to destabilise the tetragonal phase, resulting in t–m transformation at surface [5]. Therefore, it seems likely that in porous specimens with nanometric grain size (SPS-65, 90 and 120); the diffusion of water species should be more effective because of the presence of larger area of grain boundaries and of larger free surface in contact with water. In spite of that, no degradation was detected in these specimens after ageing, in contrast with the large monoclinic content measured in dense specimens, but with grain size larger than 300 nm. At the same time, transformation under stress, as observed in the grinding experiments, did not produce a clear indication of the extent of this effect in specimens with different grain size. However it seems that transformation under stress becomes more difficult in dense specimens as the grain size decreases (compare fracture toughness of SPS-120 and SPS-300).

According to Lange, F.F. [4] the change of total free energy (Δ*G_t_*_−*m*_*)* for the t-m transformation of a tetragonal particle embedded in an infinite matrix can be expressed as:

(6) $$\Delta G_{t-m}\;=\;\Delta G_{c}\;+\;\Delta U_{se}\;+\;\Delta U_{s}$$

where *ΔG_c_* (<0) is the difference in chemical free energy between the tetragonal and monoclinic phases. *ΔU_se_* (>0) is the change in elastic strain energy associated with the transformation and *ΔU_s_* (>0) is the change in energy associated with the formation of new interfaces when the transformation occurs. This will happen when:

(7) $$-\Delta G_{c}\;>\;\Delta U_{se}\;+\;\Delta U_{s}\;$$

By decreasing the number of vacancies near the surface, the driving force for t-m transformation increases, that is, (-*ΔG_c_* increases), ΔU_se_ will increase less than in the bulk, since at the surface deformation perpendicular to the surface is not restrained. The strain energy term is clearly revealed by the fact that free tetragonal powder has higher resistance to LTD than sintered bodies with tetragonal microstructure and with grain size near to the crystallite size of the powder [6]. According to Schubert and Frey [6], the strain energy contribution has a dominating influence over the surface term and it is considered to be the result of a term related to the volume increase that accompanies transformation and makes it more difficult, and another term associated to the residual stresses present in the manufactured body and to those that may be created by the diffusion of water species inside the material. Apparently, the water radicals lead to a change in lattice parameters which shows basically a lattice contraction of a tetragonal unit cell. This contraction due to proton penetration leads to a greater energy difference in the strain energy term between t- and m-phase which has a destabilising effect because it leads to a smaller activation barrier for transformation. There is also the possibility that the surface energy change *ΔU_s_* is lower in the presence of moisture or water vapour pressure. It is expected that decreasing the grain size has no important effect on the strain energy, but increases the surface term of equation (6) so that transformation is more difficult. The surface term, *ΔU_s_*, leads to a dependence of the activation barrier for t-m transformation on the particle size. Therefore, the activation barrier to form a critical nucleus is decreased by an increase in particle size. This effect qualitatively explains the dependence of amount of transformation on grain size [35].

If degradation was induced only by a change in the free energy because of the removal of vacancies near the surface, its effect would be stronger in the smaller grain sizes which have larger area of grain boundaries and of contact with water.

The stability of a grain depends also on internal stresses, which themselves depend on the level of anisotropy of the thermal expansion. For 2Y-TZP the shear stresses are larger than in 3Y-TZP because of higher thermal expansion anisotropy [36]. It is also found that larger grains contain high stresses in the area close to the grain edge compared to smaller grains [37]. Starting from the same level, the stresses decrease more rapidly for smaller grain sizes. Additionally, from Figure 2 it can be observed that the grains appear with round edges in samples SPS 65, 90 and 120. The stress level in grains with round edges is lower than in perfectly sharp-edged grains [37]. Therefore, we believe in our materials both the grain size and shape were the main contributors for maintaining a stable tetragonal phase during ageing.

Grinding in zirconia ceramics induces a surface layer with compression residual stresses and where t-m transformation has taken place [38,39]. The amount of transformation on the ground surface was used here to compare the relative transformability under stress of specimens of different grain size. Sato *et al.* [40] showed that grinding with #200 wheel produces a work affected layer which suffers a large compressive residual stresses. Kosmac *et al.* [41] reported that rough machine grinding produces reverse transformation and so the t-m transformation amounts were negligible. We have found similar results under the conditions used here; monoclinic volume fraction calculated by 2° grazing incidence was less than 10% except in very porous material SPS-65 in which it is 15%. But by using XRD with Bragg-Brentano symmetric-geometry at incidence angle 12°, the amount detected becomes negligible.

## 5. Conclusions

Stability of spark plasma sintered 3Y-TZP ceramics has been investigated under hydrothermal degradation and grinding. Materials with grain sizes less than 300 nm were highly resistant to hydrothermal degradation for the conditions studied here. Porosity has no effect on degradation in nanometric grain size materials. Severe degradation was observed in materials with grain sizes equal or larger than 300 nm. High activation barrier due to smaller grain size and less residual stress levels are considered the main causes for the high resistance to degradation. Grain size has no clear effect on transformation by grinding because a similar small amount of transformation is found in all materials, at least for the grinding conditions used here.
